# Retraction Note: Angelica sinensis polysaccharide prevents mitochondrial apoptosis by regulating the Treg/Th17 ratio in aplastic anemia

**DOI:** 10.1186/s12906-022-03752-5

**Published:** 2022-10-20

**Authors:** Zetao Chen, Li Cheng, Jing Zhang, Xing Cui

**Affiliations:** 1 Department of Gerontology, Affiliated Hospital of Shandong University of Traditional Chinese Medicine, 250014 Jinan, China; 2 Department of Acupuncture, Affiliated Hospital of Shandong University of Traditional Chinese Medicine, 250014 Jinan, China; 3 Department of Science and education, Shandong Mental Health Center, 250014 Jinan, China; 4 Department of Hematology, Affiliated Hospital of Shandong University of Traditional Chinese Medicine, 16369 Jingshi Road, 250014 Jinan, China

**Retraction Note: BMC Complement Med Ther 20, 192 (2020)**

10.1186/s12906-020-02995-4

The Editor has retracted this article. After publication, the authors became aware that the histology images in Fig. 1 C were not representative, and requested a Correction. Further assessment of the data by the Journal identified the following concerns:

- In Fig. 1D, there appears to be overlap between the NC and medium dose images.
- In Fig. 3 F, there appears to be overlap between model control and low dose images.
- In Fig. 3G, the resolution of the flow cytometry plots appears to be inconsistent. Specifically, the data point size in the medium group is larger than the other groups.
- Western blots in Fig. 3 H are presented in unnatural-looking backgrounds, with straight vertical and horizontal edges on some of the bands.
- Western blots in Fig. 3I appear to contain duplicated areas between the P38 and p-P38 images.

The authors have provided some of the raw data to address these concerns, which contained further cases of overlap or did not support the presented results. Additionally, the authors have stated that part of the data were generated by a third party. The Editor therefore no longer has confidence in the data presented in this article.

All authors have agreed to this retraction but not to the wording of this retraction notice.

